# Determinants of pneumonia among under-five children at Hiwot Fana specialized hospital, Eastern Ethiopia: unmatched case-control study

**DOI:** 10.1186/s12890-023-02593-3

**Published:** 2023-08-09

**Authors:** Mokanint Kifle, Tesfaye Assebe Yadeta, Adera Debella, Ibsa Mussa

**Affiliations:** 1 School of Public Health, College of Health and Medical Sciences, Haramaya University, Harar, Ethiopia; 2 School of Nursing and Midwifery, College of Health and Medical Sciences, Haramaya University, Harar, Ethiopia

**Keywords:** Determinants, Pneumonia, Under-five children, Case-control, Ethiopia

## Abstract

**Background:**

Globally, pneumonia is a serious public health issue. Clear evidence is necessary for the early detection and treatment of pneumonia's causes. Yet, there is limited data on this issue in the current study area. Thus, this study aimed to pinpoint the determinants of pneumonia among under-five children at Hiwot Fana Specialized Hospital, Eastern Ethiopia.

**Methods:**

A hospital-based unmatched case-control study was conducted among a sample of 348 (116 cases and 232 controls) children at Hiwot Fana Specialized Hospital from October 1 to November 30, 2022. A consecutive sampling technique was employed, and data were collected with a pre-tested interviewer-administered questionnaire. The data was entered into Epi-Data version 3.1 and analyzed using SPSS version 25 software. Bivariate and multivariate binary logistic regression analyses were fitted. Variables with a 95% confidence interval having a *p*-value < 0.05 were considered statistically significant.

**Results:**

An overall total of 347 (115 cases and 232 controls) among under-five children was included in this study. Factors such as hand washing before child feeding [AOR: 3.11 (1.74-5.57)], birth to 6 months breastfeeding [AOR: 2.76 (1.35-5.25)], zinc supplementation [AOR: 2.5 (1.33-4.40)], diarrhea in the last 2 weeks [AOR: 4.7 (2.64-8.33)], and Upper Respiratory Tract Infections in the last 2 weeks [AOR: 5.46 (3.21-10.92)] were found to be determinants of pneumonia.

**Conclusions:**

This study pointed out that the under-five pneumonia was relatively large. Factors such as hand washing before child feeding, birth to 6 months of breastfeeding, zinc supplementation of the child, diarrhea in the last 2 weeks, and Upper Respiratory Tract Infections in the last 2 weeks were determinants of under-five pneumonia. In this study, the primary risk factors for pneumonia may be preventable with no or minimal cost. Therefore, we advise suitable and sufficient health education addressing the prevention and management of pneumonia.

## Introduction

Globally, the leading infectious cause of mortality in children is pneumonia. In 2019, pneumonia accounted for 14% of all deaths of children under 5 years old, killing 740,180 children under the age of 5, accounting for 14% of all deaths of children under 5 years old but 22% of all deaths in children aged 1 to 5 years. While pneumonia affects children and families everywhere, deaths are highest in southern Asia and sub-Saharan Africa [1]. Most of these fatalities happened in underdeveloped nations, where access to healthcare is poor and few innovations have improved treatment in affluent nations [2]. Pneumonia is a significant contributor to morbidity worldwide, with an estimated 156 million occurrences and 14.9 million hospitalizations per year [1, 3].

The under-five child mortality rate decreased globally by about 50% between 2000 and 2019, but progress is still slower than expected, and 65 (32%) of 204 countries, primarily in sub-Saharan Africa and South Asia, are not on track to meet either Sustainable Development Goal (SDG) 3.2, which is to end preventable deaths of newborns and under-5 to reduce newborn mortality to at least as low as 12 per 1000 live births in every country, or reduce under-5 mortality to at least as low as 25 per 1000 live births in every country to be achieved by 2030 [4]. Nonetheless, pneumonia-related morbidity and mortality continue to be a public health issue in developing countries. Sub-Saharan Africa had the highest under-five mortality rate, with an average under-five mortality rate of 172 deaths per 1,000 live births. Pneumonia is a major cause of morbidity and mortality among under-five children in sub-Saharan Africa [5].

In Ethiopia, next to diarrhea, which causes 33% of all pediatric fatalities, pneumonia causes 28% of them. Malaria, tetanus, sepsis, prematurity, meningitis, and asphyxia together cause the remaining 8%, 7%, 5%, 5%, and 4% of pediatric fatalities, respectively [6]. In order to eliminate avoidable child deaths from pneumonia and diarrhea by 2025, the WHO and UNICEF created the integrated Global Action Plan for Pneumonia and Diarrhea (GAPPD) [7]. In addition, the Federal Ministry of Health of Ethiopia (FMOH) has incorporated the pneumococcal conjugate vaccine in its expanded program on immunization since 2011 to prevent severe forms of pneumococcal disease in childhood [8]. Despite national and international efforts, pneumonia is still a major issue in Ethiopia.

Research has revealed that, in addition to causing death, pediatric pneumonia may have significant economic consequences for the community it affects. This is mainly true for a child born in a rural setting, into a low-income family, or to a woman who has not had a basic education; they are more likely to die before turning five. Although under-five mortality has steadily decreased over the past 20 years, there have been few advancements in lowering newborn mortality [9–12].

Previous research has shown that some of the most prevalent risk factors for pneumonia include not exclusively breastfeeding, indoor air pollution, parental cigarette smoking, malnutrition, common co-morbid conditions, living in a crowded home, keeping domestic animals inside the main house, cooking with charcoal, being older at birth, having previously had an upper respiratory infection, having more than four family members, not taking zinc supplements, and not having a separate kitchen [13–21].

The contributing factors need to be studied so as to better inform and educate policymakers, programmers, implementers, and the general public about the issue. The relevant variables must be researched. Regarding the causes of pneumonia in the current research area, there is scant data. Consequently, the aim of this study was to determine what factors led to pneumonia in 2–59 month-old children at Hiwot Fana Specialty Hospital in Eastern Ethiopia.

## Methods and materials

### Study design, setting and period

A hospital-based unmatched case-control study was conducted from October to November 2022 among 347 patients (115 cases and 232 controls) at Hiwot Fana Specialized University Hospital, Harari People Regional State, eastern Ethiopia. This study site is located 517.2 kilometers east of Addis Ababa, Ethiopia. HFSUH is currently used as a teaching and referral hospital and provides different services for approximately 5.8 million people around Harar and neighbouring regions like Oromia Regional State, Dire Dawa Administrative Council, and Ethiopia Somali Regional State. The hospital has four major departments and six minor departments. The Department of Pediatrics is one of the major departments and has six units, which include the Pediatric Intensive Care Unit, Ward, Nutritional Rehabilitation Unit, Neonatal Intensive Care Unit, Outpatient Department, and Chronic Follow-Up (Source: verbal communication with the hospital HMIS head).

### Population and eligibility criteria

Children under the age of five attending Hiwot Fana Specialized University Hospital for service during the study period were considered the source population, whereas under-five children who visited the Outpatient Department (OPD) in Hiwot Fana Specialized University Hospital during the data collection period were regarded as the study population. The cases were children aged two months to five years positive for pneumonia as defined by the World Health Organization (WHO) Integrated Management of Childhood Illness (IMNCI) guideline adopted by the Ethiopian Government, and children attending under five outpatient departments of the hospital who are confirmed and recorded as having pneumonia by a physician were enrolled as cases, whereas all under five children diagnosed with non-pneumonia cases at the same outpatient department were enrolled as controls and included in the study [22, 23], whereas children aged 2–59 months whose mothers or caregivers were seriously unwell or unable to communicate were not included in the study.

### Sample size determination and sampling procedures

The sample size was calculated by the statistical  module of Epi-Info version 7.1.5.2 software using the double population proportion formula for an unmatched case-control study with the following assumption: 95% CI, 80% power of the study, the proportion of exposed cases (p1), the proportion of exposed controls (p2), and the case-to-control ratio of 1:2 were used. First, four important independent determinants (Previous history of asthma in childhood, upper respiratory tract infection two weeks ago, Use of wood for cooking, and wasting) of pneumonia among children aged under five years were considered to calculate the sample size, and those factors were taken from the previous study conducted in our country on the same study population. Finally, the factor that gives the largest sample size was used to determine the final desired sample size for the study. Thus, the largest sample size was taken based on the above assumption of wasting and was selected using the proportion of exposure among cases of 40.7%, the proportion of exposure among controls of 24.5, a ratio of control to case 2, 95% CI, 80% power of the study, and then considering the 10% non-response rate, the final sample would be 347 (case: 115 and control: 232).

A sequential and systematic random sampling was used to select cases and controls for this study. Based on the HMIS registration book, the total estimated number of under-five children from outpatient department visits for the last six months was 4,900. Of these, 700 had pneumonia under the age of five. Considering the average number of cases of pneumonia per month (700/6 = 116), all cases were taken for the study as a case. This means all under-five pneumonia cases who visited under-five outpatient departments during the data collection were included in the study. But the total estimated number of controls for the last six months before data collection was 4200 (4900–700). Dividing total controls by six months, the average number of controls per month is 4200/6 = 700, and the sampling fraction of the control is equal to (k = 700/232 = 3), meaning every third member of the control was selected by lottery method, a random number from 1 to 3 was selected to recruit the first control. Finally, interviewing the mothers and caregivers was carried out for each control and case separately.

### Data collection methods

The questionnaire was developed after reviewing related works of different literature, and some of the questionnaires were adopted from previous studies and contextualized accordingly. The questionnaire contained socio-demographic characteristics, environmental and home-related factor questions, child care factor-related questions, and pre-existing child illness-related issues. Some of the child pneumonia-related data was extracted from the document review.

Data was collected using a pre-tested and structured questionnaire. Primary data was collected through face-to-face interviews based on the questionnaire conducted with the parents of the children who were cases and controls recruited into the study. Information concerning the patient's diagnosis based on the assigned physician, the patient’s weight or height, weight or age, the child’s malnutrition status, the child’s HIV status (reactive or non-reactive), and the child's previous asthma was collected from the patient's patient chart or register. Data were collected by two Nurses (first-degree holders) working in the under-five outpatient department, and one physician and principal investigator supervised the entire data collection process.

### Variables and their measurement

#### Pneumonia

Diagnosis determined by the assigned physician and recorded based on the Federal Democratic Republic of Ethiopia Ministry of Health Integrated Management of Newborn and Childhood Illness used as a case during data collection [24].

#### Preceding 2 week of URTI

A child who had a history of an ear infection, common cold, tonsillitis, or pharyngitis in the last fifteen days before data collection [25].

#### Exclusive breastfeeding

A child who has been fed only breast milk for up to 6 months of life without other food [26].

#### Type of fuel source

Fuel in the form of wood, charcoal, electricity, animal dung, and other biomass fuels used for cooking [27].

#### Overcrowding

Is determined by the number of family members per room. A child who was not found to meet the following standard was considered to be staying in an overcrowded house. 1 room for 2 persons, 2 rooms for 3 people, 3 rooms for 5 people, 4 rooms for 7 persons, and 5 or more rooms for 10 persons [28].

### Data quality control

Data collectors and supervisors were trained for two days on the objectives, interviewing techniques, and procedures of the data collection. The questionnaire was prepared in the English language, translated to the local languages (Afaan Oromo and Amharic) to make data collection simple, and returned to English to check for consistency. It was pretested on 10% of the sample at Haramaya Hospital before the actual data collection and was revised before the data collection. Based on the results of the pretest, modifications were made to the data collection tool, and the average time required for the interview was decided. The data were checked for completeness by supervisors and investigators throughout data collection. A clerk double-entered two data sets, and the consistency of the entered data is cross-checked by comparing two separately entered data sets.

### Data processing and analysis

First, the collected data were coded, cleaned, and checked for completeness and consistency. The collected data were entered, compiled, and analyzed using EpiData version 3.1 for data entry and Exported to SPSS version 25 for analysis. Descriptive and summary statistics were conducted and reported using some tables and figure. Independent variables having a *p*-value <0.25 in the bivariable logistic regression analysis were included in a multivariable logistic regression model to assess their association with the dependent variable with a crude odd ratio with 95% CI. Hosmer-Lemeshow statistics and the variance inflation factor (VIF) were used to assess the goodness-of-fit of the models and multi-collinearity, respectively. The model was considered a good fit since it was found to be non-significant for the Hosmer-Lemeshow statistic (*p* > 0.05). A multi-collinearity test was conducted to see the correlation between independent variables using the variance inflation factor (VIF), and no variables were observed out of the VIF range of 1.011 to 5.093, which is less than 10, indicating the non-existence of multi-collinearity among the variables in this study. Finally, variables associated with the outcome variables with a *p*-value <0.05 in the adjusted analysis were considered statistically significant. The degree of association between independent and dependent variables was assessed using an adjusted odds ratio with a 95% confidence interval. AOR with a 95% CI at a *p*-value <0.05 was considered a statistically significant association with pneumonia in under-five children.

## Results

### Socio-economic and demographic characteristics

Out of 347 (115 cases and 232 controls) children who were approached for an interview, there was a response rate of 100%. The mean ages of the mothers were 28.26 years (SD ± 6.8) for controls and 27.9 years (SD ± 6.4) for cases, whereas the mean ages of the children were 16.76 months (SD ± 13.9) for cases and 19.73 months (SD ± 15.8) for controls, respectively. In the sampled population, 73 (63.5%) of the cases and 134 (57.8%) of the controls were above the age of 25. In terms of gender, 132 (56.9%) of the control group and 61 (53%) of the case group were men. Around 110 (47.4%) of the controls and 70 (60.9%) of the cases lived in rural areas. More than 60% of children, both in cases and controls, were male. Regarding the educational status of mothers, 46.1% of cases and 39.2% of controls were illiterate. Similarly, most of the partners in the cases (35.7% of cases) were illiterate compared with the partners in the controls, who were 24.6%. Concerning marital status, 93.9% of cases and 92.7% of controls were married **(**Table 1**).**

**Table 1:** Socio-demographic characteristics of participants at Hiwot Fana specialized hospital, Eastern Ethiopia, 2022 (*n* = 115 cases & 232 controls)

**Variables**	**Categories**	**Pneumonia**	
		**Case n(%)**	**Control n (%)**
Age of child (months)	<12 months	63 (54.8)	113 (48.7)
	>=12 months	52 (45.2)	119 (51.3)
Sex of child	Male	61(53.0)	132 (56.9)
	Female	54 (47.0)	100 (43.1)
Residence	Rural	70 (60.9)	110 (47.4)
	Urban	45 (39.1)	122 (52.6)
Age of mother (years)	<25 years	42 (36.5)	98 (42.2)
	>=25 years	73 (63.5)	134 (57.8)
Marital status of parents	Married	108 (93.9)	215 (92.7)
	Widowed	2 (1.7)	4 (1.7)
	Divorced	5 (4.3)	13 (5.6)
Mother’s educational level	Illiterate	53 (46.1)	91 (39.2)
	able to read and write	5 (4.3)	9 (3.9)
	Primary	34 (29.6)	77 (33.2)
	secondary and above	23 (20.0)	55 (23.7)
Mother’s occupation	House wife	73 (63.5)	142 (61.2)
	Merchant	18 (15.7)	23 (9.9)
	daily laborer	6 (5.2)	23 (9.9)
	Student	5 (4.3)	7 (3.0)
	Others*	0 (0.0)	5 (2.2)
	Government employee	13 (11.3)	32 (13.8)
Father’s educational level	Illiterate	41 (35.7)	57 (24.6)
	able to read and write	4 (3.5)	8 (3.4)
	Primary	36 (31.3)	69 (29.7)
	secondary and above	34(29.6)	98 (42.2)
Father’s occupation	Farmer	59 (5.13)	96(41.4)
	Merchant	13(11.3)	36(15.5)
	daily laborer	89(77.4)	29(12.5)
	Student	3(2.6)	1(0.4)
	Others*	8(7.0)	18(7.8)
	Government employee	24(20.9)	52(22.4)

Others*Drivers, private employee, NGO worker

### Environmental and home-related factor of pneumonia

The majority of cases 62 (53.9%) and controls 107 (46.1%) used wood as a source of fuel for cooking, and 87 (75.7%) of cases and 180 (77.6%) of controls cooked food in the kitchen. About 82.6% of cases and 79.3% of the control’s house had a window. About 85 (73.9%) of cases and 159 (68.5%) of controls had a kitchen separated from the main house. About 72 (62.6%) of cases and 75 (32.3%) of controls had only water for hand washing. In addition, there was a statistically significant association between case and control groups in exposure to materials used for hand washing before child feeding (χ2 = 29.067, *p* = 0.000) and family member sleeping per room (χ2 = 6.601, *p* = 0.010) (Table 2).

**Table 2:** Environmental and home related factor of pneumonia under five children in Hiwot Fana Specialized Hospital, Eastern Ethiopia, 2022 (*n* = 115 cases and 232 controls)

**Variables**	**Categories**	**Pneumonia**	
		**Case n(%)**	**Control n (%)**
Fuel material mostly used for cooking	Charcoal	30 (26.1)	61 (26.3)
	Wood	62 (53.9)	107 (46.1)
	Kerosene	15 (13.0)	47 (20.3)
	Electricity	8 (7.0)	17(7.3)
Family members sleep	Overcrowding	105(91.35)	187 (80.6)
	not crowded	10 (8.7)	45 (19.4)
Does your house have window	Yes	95 (82.6)	184 (79.3)
	No	20 (17.4)	48 (20.7)
Windows daily opening	Yes	80 (69.6)	158 (68.1)
	No	35 (30.4)	74 (31.9)
Hand wash before child feeding	only water	72 (62.6)	75(32.3)
	water and ash	1 (0.9)	2(0.9)
	water and soap	42(36.5)	155(66.8)
Mainly used cooking room	living room	22 (19.1)	35 (15.1)
	Kitchen	87 (75.7)	180 (77.6)
	Outdoors	6 (5.2)	17 (7.3)
Kitchen separated from main house	Yes	85 (73.9)	159 (68.5)
	No	30 (26.1)	73 (31.5)
Kitchen windows Opening daily	Yes	49 (42.6)	121 (52.2)
	No	66 (57.4)	111 (47.8)
Child stays during cooking	Hold on my back	38 (33.0)	73 (31.5)
	Out cooking room	77 (67.0)	159 (68.5)
Cigarette smoking family member	Yes	25 (21.7)	69 (29.7)
	No	90 (78.3)	163 (70.3)

**Fig. 1:**
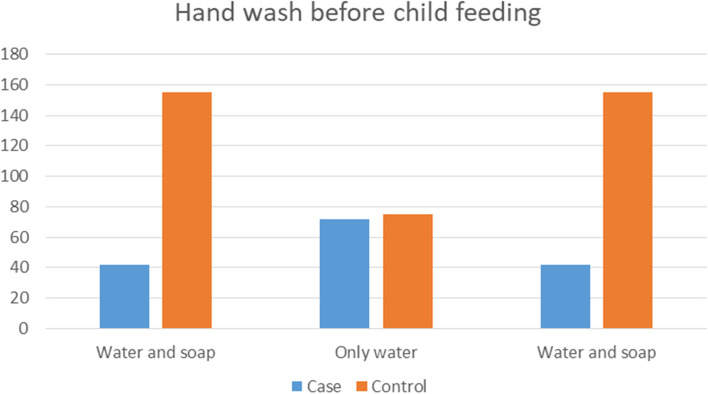
Hand wash before child feeding practice of participants in at Hiwot Fana specialized hospital, Eastern Ethiopia, 2022 (*n* = 115 cases & 232 controls)

Regarding hand washing before child feeding, about 72 (62.6%) of cases and 75 (32.3%) of controls used only water for hand washing (Fig. 1).

### Nutritional and child care related factor to pneumonia

Regarding the children's immunization status, 124 (53.4%) of controls and 42 (36.5%) cases were fully immunized, whereas 36 (31.3%) cases and 80 (34.5%) controls were immunized just to the age at which they should have been immunized, and the remaining children were unvaccinated, which was 37 (32.2%) of cases and 28 (12.1%) of controls. In terms of the children's nutritional health, more than half—79 (68.7%) of cases and 203 (87.5%) of controls—were exclusively breastfed. There was a statistically significant difference between case and control groups in vaccination status (χ2 = 21.43, *p* = 0.000), exclusive breastfeeding of the child (χ2 = 17.9, *p* = 0.000), and place of delivery (χ2 = 6.870, *p* = 0.032). A total of 24 cases (20.9%) and 61 controls (26.3%) were wasted. Of the cases, 23, or 20.0%, and the controls, 55, or 23.7%, were both underweight. Forty-one (35.7%) cases and fifty-seven (26.4%) control children were delivered at home. Around 104 (89.6%) of the cases and 201 (86.6%) of the control groups had parents who took care of the kids at home **(**Table 3**).**

**Table 3:** Nutritional and child care related factor to pneumonia among under five in Hiwot Fana Specialized Hospital, Eastern Ethiopia, 2022 (*n* = 115 cases and 232 controls)

**Variables**	**Categories**	**Pneumonia**	
		**Case n(%)**	**Control n (%)**
Vaccination status	fully vaccinated	42 (36.5)	124 (53.4)
	Up-to-date	36 (31.3)	80 (34.5)
	Unvaccinated	37 (32.2)	28 (12.1)
Breast feeding practice (birth to 6 months)	Exclusive breast feeding	79 (68.7)	203 (87.5)
	Mixed	36 (31.3)	29 (12.5)
Vitamin A supplementation	Yes	72 (62.6)	142 (61.2)
	No	43 (37.4)	90 (38.8)
Zinc supplementation	No	49 (42.6)	80 (34.4)
	Yes	66 (57.4)	152(65.5)
Weight for height (WFH)	Wasted	24(20.9)	61 (26.3)
	Normal	91 (79.1)	171 (73.7)
Weight for age (WFA)	Underweight	23(20.0)	55 (23.7)
	Normal	92 (80.0)	177(76.3)
Place of delivery	Home	41 (35.7)	57 (24.6)
	public institution	73 (63.5)	165 (71.1)
	private institution	1 (0.9)	10 (4.3)
Child’s care giver at home	Parental care	103 (89.6)	201 (86.6)
	Home care giver	12 (10.4)	31 (13.4)

### Child’s pre-existing illness related factor

Concerning pre-existing illness-related factors in children, 65 (56.5%) of cases and 45 (19.4%) of controls had a history of diarrhea for the last two weeks prior to the data collection period. Fifty-three (46.1%) of cases and 30 (12.9%) of controls had a history of Acute Upper Respiratory Tract Infections (AURTI) prior to the data collection period. In addition, both the presence of diarrhea (χ2 = 48.95, *p* = 0.000) and Upper Respiratory Tract Infections (URTI) (χ2 = 46.45, *p* = 0.000) in the last two weeks for the child were statistically significant differences between the case and control groups. About 111 (96.5%) of cases tested for HIV, and 2 (1.7%) of them were reactive (zero-positive), while 222 (95.7%) controls tested for HIV, and 6 (2.8%) of them were reactive (seropositive). Thirty-seven (33% of cases) and sixty-three (27.2%) of controls had a history of malnutrition. Only 4 (4.3%) of cases and 11 (4.7%) of control children had a history of asthma (Table 4).

**Table 4:** Childs pre-existing illness related factor to pneumonia among under five children in Hiwot Fana Specialized Hospital, Eastern Ethiopia, 2022 (*n* = 115 cases and 232 controls)

**Variables**	**Categories**	**Pneumonia**	
		**Case n(%)**	**Control n (%)**
Malnutrition for the child	Yes	37 (33.0)	63 (27.2)
	No	78 (67.0)	169 (72.8)
HIV tested	Yes	111 (96.5)	222 (95.7)
	No	4 (3.5)	10 (4.3)
If yes, test result	Reactive	2(1.7)	6 (2.6)
	non-reactive	113(98.3)	226 (97.4)
URTI Diarrhea in the last 2 weeks	Yes	65 (56.5)	45 (19.4)
	No	50 (43.5)	187 (80.6)
A URTI in the last 2 weeks	Yes	53 (46.1)	30 (12.9)
	No	62 (53.9)	202(87.1)
History asthma for child	Yes	4 (4.3)	11 (4.7)
	No	111 (96.5)	221 (95.3)

### Determinants independently associated with under-five pneumonia

In the unadjusted analysis, a total of 13 variables were identified in the bivariate analysis with *p*-values less than 0.25 and selected for additional investigation. Accordingly, rural residence (COR=1.73,95%, CI:1.10-2.72), illiterate mother (COR=2.07,95%, CI:1.1-9.63), father occupation, overcrowding home (COR=2.26,95%, CI:1.22-5.22), only water used to wash hands before feeding children (COR=3.54,95%, CI:2.21-5.69), not opening kitchen window daily (COR=1.47,95%, CI:0.94-2.30), malnutrition (COR=1.32,95%, CI:0.85-2.15), unvaccinated child (COR=3.90,95%, CI: 2.14-7.13), had not exclusively breastfeeds first six months (COR=3.19,95%, CI:1.83-5.55), supplemented with zinc (COR=1.41,95%, CI:0.89-2.23), home delivery (COR=7.19,95%, CI:0.89-58.41), that child had diarrhea in the previous two weeks (COR=5.40,95%, CI:3.30-8.83), and that child had Acute Upper Respiratory Tract Infections in the previous two weeks (COR=5.76,95%, CI:3.39-9.79) were associated to pneumonia in children under the age of five **(**Table 5**).**

**Table 5:** Multivariable logistic regression model for factors associated with under-five pneumonia, Hiwot Fana Specialized Hospital, Eastern Ethiopia, 2022

Factors	Categories	Pneumonia		COR (95% CI)	AOR (95% CI)
		Case	Control		
Residence of child	Rural	70	110	1.73 [1.10-2.72]*	1.25[0.37-4.03]
	Urban	45	122	1	1
Educational level of father	Illiterate	41	57	2.07[1.19-3.63] *	2.14[0.65-7.08]
	Able to read and write	4	8	1.44[0.41-5.09]	1.12[0.17-7.44]
	Primary	36	69	1.50[0.86-2.64]	2.08[0.78-5.56]
	Secondary and above	34	98	1	1
Occupation of father	Farmer	59	96	1.33[0.74-2.38] *	0.52[0.23-1.22]
	Merchant	13	36	0.78[0.35-1.74]	0.57[0.22-1.46]
	daily laborer	8	29	0.60[0.24-1.50]	0.38[0.12-1.21]
	Student	3	1	6.50[0.642-65.76]	9.39[0.57-154.01]
	Others	8	18	0.96[0.37-2.52]	0.48[0.14-1.60]
	Government employee	24	52	1	1
Family members sleep	overcrowding	105	187	2.53[1.22-5.22] *	1.70[0.67-4.32]
	not crowded	10	45	1	1
Hand wash before child feeding	only water	72	75	3.54[2.21-5.67] *	3.11[1.74-5.57 **
	water and ash	1	2	1.85[0.16-20.85]	3.10[0.17-56.52]
	water and soap	42	155	1	1
Opening kitchen windows daily	No	66	111	1.47[0.94-2.30]	0.81[0.42-1.54]
	Yes	49	121	1	1
Malnutrition	No	38	63	1.32[0.82-2.15] *	0.74[0.37-1.47]
	Yes	77	169	1	1
vaccination status of child	fully vaccinated	42	124	1.33[0.79-2.25]	1.25[0.71-2.10]
	Up-to-date	36	80	3.90[2.14-7.13] *	2.05[0.90-5.18]
	unvaccinated	37	28	1	1
Birth to 6 months breast feeding	Mixed	36	29	3.19[1.83-5.55] *	2.76[1.351-5.250] **
	Exclusive breast feeding	79	203	1	1
Zinc supplementation of child	No	49	80	1.41[0.89-2.23] *	2.50[1.33-4.40] **
	Yes	66	152	1	1
Place of delivery	Home	41	57	7.19[0.89-58.41] *	3.14[0.31-31.68]
	public institution	73	165	4.42[0.56-35.20	4.53[0.50-41.06]
	private institution	1	10	1	1
Diarrhea in the last 2 weeks	Yes	65	45	5.40[3.30-8.83] *	4.70[2.64-8.32] **
	No	50	187	1	1
URTI in the last 2 weeks	Yes	53	30	5.76[3.39-9.78] *	5.46[3.21-10.92]**
	No	62	202	1	1

Variables significant in bivariate and multivariate at (*P < 0.25, **P < 0.05) respectively, *1*: Reference, *CI* Confidence Interval, *COR* Crude Odd Ratio, *AOR* Adjusted Odd Ratio. Others*Drivers, private employee, NGO worker

However, only five variables (hand washing material used before child feeding, exclusive breastfeeding practice, zinc supplementation of the child, presence of diarrhea in the last two weeks, and presence of AURTI in the last two weeks) had significant associations with pneumonia at *p*-values less than 0.05 in multi-variable logistic regression analysis.

The odds of developing pneumonia among children with hand washing before feeding using only water were 3.11 times more likely as compared to those with hand washing using both water and soap [AOR: 3.11 (1.74-5.57)]. Those children who did not have exclusive breastfeeding were 2.76 times more likely to develop pneumonia than those with exclusive breastfeeding [AOR: 2.76 [(1.35–5.25)]. Similarly, children who had never received zinc supplementation were 2.4 times more likely to develop pneumonia compared to their counterparts [AOR: 2.50 (1.33–4.40)].

The likelihood of pneumonia was 4.70 times higher among under-five children who had a history of diarrhea during the past fifteen days when compared to their counterparts [AOR: 4.70 (2.64-8.32)]. Those children who had Acute Upper Respiratory Tract Infections in the last 2 weeks prior to the study period were 5.46 times more likely to have pneumonia than their counterparts [AOR: 5.46 (3.21–10.92)].

## Discussions

The study was aimed at identifying determinants of pneumonia among under-five children at Hiwot Fana Specialized University Hospital in Eastern Ethiopia. This study pointed out that a total of 347 (115 cases and 232 controls) under-five children had hand washing before child feeding, birth to 6 months of breast feeding, zinc supplementation, diarrhea in the last 2 weeks, and URTI in the last 2 weeks, all of which are determinants of under-five pneumonia.

Regarding hand washing practice before feeding a child, parents who solely used water for hand washing before feeding their children had a 3.11 times higher risk of pneumonia in children under the age of five. This study is in line with research done in northern Ethiopia, where those who used only water were at higher odds of developing pneumonia than those who used both water and soap [23]. Studies conducted elsewhere revealed that proper hand washing with soap reduces the burden of pneumonia by 50% [29]. In connection with this, the WHO recommends that children and caregivers wash their hands with soap and water frequently throughout the day to lower the risk of exposure to bacteria and other microbial agents that may cause pneumonia [30].

Lack of breastfeeding is a leading risk factor for child morbidity and mortality in developing countries [31]. This study revealed that due to breastfeeding practices in the first six months of life, the odds of developing pneumonia in children who were on mixed breastfeeding increased 2.76 times as much as those who were exclusively breastfed during their birth to six-month-old duration. This result is consistent with the study conducted in developing countries, where non-breastfed children experience a 14-fold increase in all-cause mortality compared to those who are exclusively breastfed for 6 months [31–33], and Uganda, 2.9 [34]. Also, a systematic literature review and meta-analysis showed that suboptimal breastfeeding increased the risk for pneumonia morbidity and mortality outcomes across age groups [33]. A meta-analysis of 10 studies from developing and developed countries on risk factors of pneumonia identified that infants who were not exclusively breastfed were more than twice as likely to develop severe Acute Upper Respiratory Tract Infections as those who were exclusively breastfed [35]. This could be due to the fact that children who were not exclusively breastfed had a lower chance of preventing infections, as breast milk has many immunological properties that are likely to protect against infections in children.

Different studies revealed that zinc deficiency was associated with an increased risk of infection, particularly pneumonia [35]. Similarly, in the current study, it is implied that children who were never given zinc supplements had a 2.4-fold increased risk of having pneumonia. This result is consistent with a case-control study in the Kersa district of Ethiopia, which reported that children who had not ever been supplemented 1.7 times were associated with pneumonia [17]. Also, studies conducted in the US and Pakistan reported a reduction in pneumonia incidence and prevalence among children who received zinc supplementation [36, 37]. The other two studies, both from developing regions, reported a 50% inverse association between zinc supplementation and severe use of zinc in children [38, 39]. This could be due to the fact that zinc supplementation plays an important role in preventing respiratory infections in children and promoting their health due to its protective and anti-inflammatory effects [40, 41].

This study showed that a child who had a history of diarrhea during the last 2 weeks had a fourfold increased risk of pneumonia as compared to a child who did not have a history of diarrhea. This result is supported by a study from Zimbabwe, Southwest Ethiopia, and Tigray, Ethiopia [17, 42, 43]. These results were in harmony with studies conducted in Ethiopia, which report that children with a history of diarrhea have a three-fold higher pneumonia risk than their counterparts [44]. Contrary to this, a case-control study conducted in southern Ethiopia showed no association with children who had a history of diarrhea during the last 2 weeks [45]. The possible explanation could be due to the fact that having a concomitant illness like diarrhea puts children at a higher risk of contracting pneumonia by compromising their immunity.

This study’s findings also indicated that children who had Acute Upper Respiratory Tract Infections in the last two weeks were five times more likely to develop pneumonia compared with their counterparts. This was consistent with the case-control study in northwest Ethiopia, 5.33 [23], and southwest Ethiopia, 5.2 [17]. Also, the finding in the Netherlands showed a strong relationship between the occurrence of community-acquired pneumonia and an increasing number of previous AURTIs [46]. The possible explanation for this could be that Acute Upper Respiratory Tract Infections ,which include rhinitis, pharyngitis, tonsillitis, and laryngitis, increase susceptibility to infections that predispose patients to pneumonia or that there might be a descending infection from the upper to the lower respiratory tract.

Overall, the study findings imply that in Ethiopia, educating women to recognize diarrhoea and upper respiratory infections earlier to prevent the development of pneumonia and creating awareness about how exclusive breastfeeding limits the progression of pneumonia would be very important. Hence, women need to be educated in well practicing hand washing or using alcohol based sanitizer, and refraining from habit of smoking which vividly affects lungs.

### Strength of the study

The strength of the study was that we were capable of evaluating the association of pneumonia with other exposure variables since it was a case-control study. Also, it had a high response rate.

### Limitation of the study

The institution-based nature of the study could limit the generalizability of the findings. The other limitation of the study was that it relied on participants’ self-reported data, which was prone to recall bias, social desirability bias, and interviewer bias due to the retrospective tracking of information beyond the advantages of a case-control study. However, attention was given to the study procedures, including the process of training data collectors and close supervision throughout the activity to minimize the expected biases. A smaller sample size in certain categories reduced the precision of the study.

## Conclusions

This study pointed out that the under-five pneumonia was relatively large. Factors such as hand washing before child feeding, birth to 6 months of breastfeeding, zinc supplementation of the child, diarrhea in the last 2 weeks, and Upper Respiratory Tract Infections in the last 2 weeks were determinants of under-five pneumonia. In this study, the primary risk factors for pneumonia may be preventable with no or minimal cost. Therefore, we advise suitable and sufficient health education addressing the prevention and management of pneumonia.

## Data Availability

The data sets used for this study are available from the corresponding authors upon reasonable request.

## Abbreviations

AOR: Adjusted odds ratio
AURTI: Acute Upper Respiratory Tract Infections
BCG: Tuberculosis Vaccine (Bacillus Calmette-Guerin
CAP: Community-acquired pneumonia
CI: confidence interval
COR: crude odds ratio
EDHS: Ethiopian Demographic and Health Survey
EMDHS: Ethiopian Mini Demographic and Health Survey
ETB: Ethiopian Birr
FMoH: Federal Ministry of Health
GAPPD: Global Action Plan for Pneumonia and Diarrhea
HFSUH: Hiwot Fana specialized University hospital
HMIS: Health Management Information System
HIV: human immune virus
AIDS: acquired immunodeficiency syndrome
IMNCI: Integrated management Neonatal and childhood illness
OPD: Outpatient Department
PCV: Pneumococcal Conjugated Vaccine
SDG: sustainable development goal
SPSS: Statistical package for social sciences
UNFPA: United Nations Population Fund Agency
UNICEF: United Nations International Children Fund
URTI: upper respiratory tract infection
USAID: United States Agency for International Development
WHO: World Health Organization
